# Castor Oil Soap

**Published:** 1871-06

**Authors:** 


Castor Oil Soap.—It is somewhat remarkable that our present English pharmacy has no pure medicinal soap possessing any characteristic property or medical activity. The ordinary Castile soap, being that which is commonly used for that ordered by the Pharmacopœia, can scarcely be considered a satisfactory article when we consider its composition and the mode of its manufacture. Having recently had occasion to direct my attention to this subject, it occurred to me that castor-oil offered some advantages, and would yield a soap possessing qualities very desirable in an article which so frequently formed the medium or adjunct for administering other active remedies. On putting this idea into practice, I found that a soap prepared from this oil has rather marked qualities ; but my opportunities do not afford me the means of properly testing its medicinal properties. I believe that it will be found that it has sufficient aperient power to relax the bowels when taken consecutively for several days ; but I believe its greatest value will be found as an adjunct to other aperients.

This at least is the result I have arrived at. It is, of course, well known that the purgative principle of castor-oil has been ascribed by Soubeiran to the existence of a supposed oleo-resin, and that the resinoleic acid is extremely acrid. I find when the oil is saponified that this acrid principle is either entirely or partially liberated, and does not continue masked as it is in the oil in its natural state, nor neutralized, as might be expected, by the alkali. It is to this fact, I think, we must look for any active property this soap may possess; and here I must leave the matter for the further investigation of the medical and pharmaceutical professions. The physical properties of the soap are in its favor for use in medicine. It has a clean yellowish-white color, is free from smell; it soon becomes dry, hard, and is easily powdered; it has no tendency to soften or deliquesce on exposure to the air. In proof-spirit it makes a perfectly clear and colorless solution, with only a little sediment. I shall forward a specimen to the Society for the inspection of those who may feel interested.—*F. M. Rimmington, in the London Pharm. Journal.*

